# Next Steps for Elevating Health on Trade and Investment Policy Agendas: Comment on "How Neoliberalism Is Shaping the Supply of Unhealthy Commodities and What This Means for NCD Prevention"

**DOI:** 10.15171/ijhpm.2019.126

**Published:** 2019-12-07

**Authors:** Belinda Townsend

**Affiliations:** School of Regulation and Global Governance, Australian National University, Canberra, ACT, Australia.

**Keywords:** Trade and Health, Non-communicable Disease, Commercial Determinants of Health, Governance, Trade Policy

## Abstract

Despite intergovernmental calls for greater policy coherence to tackle rising non-communicable diseases (NCDs), there has been a striking lack of coherence internationally and nationally between trade and health sectors. In this commentary, I explore the arguments by Lenucha and Thow in relation to barriers for greater coherence for NCDs, apply them to regional trade agreements, and point to next steps in research and advocacy for greater attention to health and NCD prevention in government trade agendas.

## Introduction

Over the past two decades there has been an intensification of intergovernmental strategies, plans and Declarations calling for greater action to address non-communicable diseases (NCDs), mirroring increases in preventable deaths and morbidity from these diseases globally.^1^ From the first World Health Organization (WHO) Global Strategy nearly two decades ago, to successive WHO Global Action Plans, the United Nations Political Declaration on the Prevention and Control of Diseases, and the Sustainable Development Goals, NCDs have received significant global attention, albeit with mixed results for prioritising multisectoral action and coherence across policy sectors.^2^

As Lenucha and Thow^3^ point out, much of the research examining this policy incoherence has focused on the strategies of commercial and industry actors in influencing policy-making to shape their interests. Documented strategies by firms include the promotion of advertising and marketing to youth, donations to political representatives, partnering with governments, funding biased research, co-opting health professionals and policy-makers, and sponsoring campaigns to favourably influence public opinion in their interests.^4,5^

The research is telling of the economic power of industry actors to gain a seat at the table. At the Third United Nations High-Level meeting on NCDs in September 2018, for example, the Worldwide Brewing Alliance held an event promoting the role of the beer sector in advancing the Sustainable Development Goals. Meanwhile governments met to discuss action plans for targets such as 3.5 to reduce the harmful use of alcohol. Research on the strategies of influence used by industry actors and exposing their interference in global health and NCD policy-making is important, but as Lenucha and Thow^3^ rightly note, unless we understand why industry actors gain seats at the table and exert influence over government, we are less equipped to counter and ultimately shift the agenda in favour of public health and social interests including NCD prevention.

## Neoliberalism and Trade and Investment

Rather than seeing industry actors as exerting agency over policy-makers in a vacuum, Lenucha and Thow argue that the context matters, and in particular that ways of “thinking and doing” which are historically informed and embedded in contemporary institutions, matter. In examining barriers to policy coherence for NCDs, and in particular using the political economy of tobacco in African countries, Lenucha and Thow show that the neoliberal paradigm has “conditioned the policy environment in ways that promote the supply of unhealthy commodities.” This approach to examining the ‘deep core’ of neoliberalism in public policy-making^6^ points to the importance of understanding the way that structures can shape agency. I agree with the authors and have argued elsewhere that the dominant neoliberal ideas in trade policy-making – for continued export growth and private enterprise with a reduced role of the state in regulation – constrain attention to the wider social determinants of health that are shaped by trade deals.^7^

We can also see these dynamics playing out in new regional trade and investment agreements through which attention to the potential public health impacts, or coherence with government NCD targets, remain largely on the periphery. The Trans-Pacific Partnership agreement, a mega regional trade deal initially led by the United States with eleven counties in the Pacific Rim, is a key example of the new generation of trade agreements that contain a raft of measures which constrain public regulatory space and enable greater access to health-harmful products including the NCD risk factors tobacco, alcohol, and ultra-processed foods.^8^

Framing analysis of policy actors’ submissions in Australia has shown how industry actors use neoliberal framing to urge policy-makers to promote export growth and remove perceived regulatory “barriers” to trade underpinned by assumptions that the economic benefits will trickle down to all in society.^7^ These framings work because they align with what policy-makers in government trade departments see as their key objectives from decades of neoliberal reform in public policy, and thus policy-makers and industry actors share a common framing and worldview that facilitates their engagement.

This worldview is particularly antithetical to public health objectives in the trade policy domain when arguments for reducing alcohol harm, tobacco use, and consumption of ultra-processed foods, or facilitating access to generic medicines for NCD treatment, come up against alcohol, food, tobacco or originator pharmaceutical industries in countries. Lenucha and Thow show, for example, that policy-makers in African countries have supported tobacco industries not because of the power of the tobacco industry as lobbying agents, but because of their shared ideas for the role of market enterprise in facilitating economic development.

In government trade policy-making, these shared neoliberal ideas and assumptions often result in privileged access for industry actors over public health non-governmental organization (NGO) actors. During the Trans-Pacific Partnership negotiations, for example, the United States gave privileged and confidential access to sections of the draft text to more than 500 US-based corporate actors, and a small number of labour union and NGOs.^9^ In Australia, interviews with stakeholders reveal greater satisfaction by industry actors with the formal government policy processes for consultation, with public health NGO actors and academic experts lamenting a lack of meaningful engagement.^10^ These apparent power imbalances between industry and public health NGOs appear to be reflected inside government, where government Health departments appear reliant on trade officials who act as gatekeepers to treaty text.^3^

## Next Steps and Future Research

The result of years of neoliberal reforms in public policy-making worldwide is that the dominant ideas, embedded in policy institutions, favour engagement with industry actors and export promotion and trade liberalisation, creating problems for NCD coherence. How might this be overcome? The task seems daunting, and Lenucha and Thow argue that one approach is to focus on the oft-neglected supply side, and in the context of tobacco and agricultural production, approaches that promote healthy product environments through engaging with agribusiness.

In examining trade policy, I have documented with colleagues how the strength of exporter interests over a particular product appears to shape government’s willingness to consider the health implications in their trade mandates.^3^ Australia for example has weak tobacco exports, is strong on domestic tobacco control, and informants have reported greater attention to the potential consequences for tobacco control regulation in trade deals.^3^ In contrast, as a major alcohol exporter, informants have reported significant barriers to elevating attention to alcohol harm and alcohol regulatory consequences in Australia’s trade policy processes. The hypocrisy has been pointed out by other scholars, and was particularly stark when Australia was defending its tobacco legislation at the World Trade Organization while simultaneously raising trade concerns with Thailand and other countries over their proposed alcohol labelling regimes.^11^ To tackle supply would be to encourage alcohol exporters to change their products to truly healthy ones, a task that would help reduce export pressures on governments, but which also seems daunting.

Another approach, which can be pursued in tandem with advocating healthy product supply, is to reframe the economics of these health-harmful industries which contribute significant costs through increasing NCD morbidity and mortality. Further research on the causal links between trade and investment agreements and harmful products, such as those detailing increased consumption of sugary drinks,^12^ could help make the connection for public health oriented policy-makers. Further, developing costings of the economic impact of these diseases on the health system and on pharmaceutical expenditures, could help reframe the economics of trade to account for the true costs of facilitating health-harmful products.

Research that explores examples of success and their lessons for advocacy could also enable greater understanding of how to elevate health above profit motives. In 2003, for example, Australia banned the manufacturing and importation of asbestos, a product used in building materials but which was classed as a health hazard due to links between inhaling fibres and cancer. Part of this story is shifting supply to other forms of building material. Another is strong concerted advocacy by an international trade union movement of workers, supported by health experts and associations. The role of the union movement, and of workers willing to transition away from employment in health-harmful industries, including coal, requires coalition building by public health advocates, and strong government support for just transitions and secure and safe work.

Other examples outside of the NCD domain are also revealing of how coalitions can shift supply. Workers in a formerly coal dominated area in the La Trobe Valley of South-eastern Australia have established a successful worker cooperative to supply renewable energy products to the region.^13^ Could workers, industry and governments contribute to a supply shift in heath-harmful products? Cases such as these are worth exploring for identifying the framing strategies and coalitions of actors, including industry allies where appropriate, that have formed in other sectors and have enabled the elevation of social and health interests above profit.

## Ethical issues

Not applicable.

## Competing interests

Author declares that she has no competing interests.

## Author’s contribution

BT is the single author of the paper.
